# Changes in the chronic and postprandial blood lipid profiles of trained competitive cyclists and triathletes following a ketogenic diet: a randomized crossover trial

**DOI:** 10.1186/s13102-023-00801-5

**Published:** 2024-01-16

**Authors:** Austin J. Graybeal, Andreas Kreutzer, Kamiah Moss, Meena Shah

**Affiliations:** 1 School of Kinesiology & Nutrition, College of Education and Human Sciences, University of Southern Mississippi, 39406 Hattiesburg, MS USA; 2 Department of Research Data Science & Analytics, Cook Children’s Health Care System, 76104 Fort Worth, TX USA; 3 Physical Medicine and Rehabilitation, Baylor Institute for Rehabilitation, 75246 Dallas, TX USA; 4 Department of Kinesiology, Harris College of Nursing and Health Sciences, Texas Christian University, 76129 Fort Worth, TX USA

**Keywords:** Ketogenic diet, Blood lipids, Cholesterol, High-carbohydrate diet, Endurance athletes, High-fat diets, Low-carbohydrate diets

## Abstract

**Background:**

The ketogenic diet (KD) is the most popular carbohydrate restriction strategy for endurance athletes. However, because the primary goal of employing the KD is to gain a competitive advantage in competition, endurance athletes may be less concerned with the influence of the KD on their cardiometabolic health; particularly their blood lipid profiles. Thus, the purpose of this study was to examine the chronic and postprandial blood lipid alterations following a two-week ad libitum KD compared to an ad libitum high-carbohydrate diet (HCD) and the athletes’ habitual diet (HD) in a group of trained competitive cyclists and triathletes.

**Methods:**

Six trained competitive cyclists and triathletes (female: 4, male: 2; age: 37.2 ± 12.2) completed this randomized crossover trial, which required them to follow a two-week ad libitum KD and HCD in a randomized order after their HD. Fasting blood lipids were collected following their HD and after two-weeks of the KD and HCD conditions. Postprandial blood lipid responses to a test meal reflective of the assigned diet were collected at the end of each diet condition.

**Results:**

Fasting total cholesterol (TC) was significantly higher following the KD compared to the HD (*p* < 0.001) and HCD (*p* = 0.006). Postprandial incremental area under the curve for triglycerides (TRG), TRG:HDL ratio, and VLDL-C were significantly higher following the KD test meal compared to the HD (all *p* < 0.001) and HCD (all *p* = 0.001) test meals but LDL-C and LDL:HDL ratio were significantly lower following the KD compared to the HD and HCD test meals (all *p* < 0.001).

**Conclusions:**

Trained competitive cyclists and triathletes demonstrate increased TC in response to a two-week KD compared to a HCD or HD. Endurance athletes contemplating a KD should consider the potential for these blood lipid alterations, and future research should focus on postprandial blood lipid responses to determine if these changes manifest in chronic blood lipid shifts.

**Trial registration:**

ClinicalTrials.gov Identifier: NCT04097171 (11 October 2019).

## Background

Endurance athletes regularly alter their habitual dietary patterns during periods of intensive training to enhance performance [1, 2]. Although the recommendations of several professional organizations suggest that high-carbohydrate diets (HCD) are more effective for supporting endurance performance, the popular dietary strategies endurance athletes employ in practice are often diametrically opposed to these recommendations; where it is common for endurance athletes to participate in short- or long-term carbohydrate restriction to elicit specific metabolic adaptations. The ketogenic diet (KD) is unequivocally the most popular carbohydrate restriction strategy for endurance athletes; it is defined as a very-low carbohydrate diet (< 10% of total daily energy from carbohydrates [3]) aimed at achieving ketosis [4]—the metabolic condition in which elevated ketone bodies shift substrate utilization toward a reliance on fat in situations where it would typically use glycogen [1, 2]. Ketosis can be achieved in as few as 3–5 days of following a KD [5, 6], and prior reports have suggested that achieving a “fat-adapted state” offers several endurance performance benefits [4, 7]—the primary theory for pursuing the KD despite its opposition to professional guidelines. As such, when combined with the well-reported effectiveness for both weight reduction and cardiometabolic health improvements in the general population [4, 8], it is unsurprising that the KD is considered to be both *healthy* and *effective* amongst endurance athletes.

While the realized endurance performance benefits of a KD in practice remains an area of contention, the cardiometabolic health benefits of a KD are commonly misperceived by endurance athletes in practice. For example, it is often assumed that the reported benefits of a KD are universal despite the fact that the majority of observed improvements have occurred in individuals with overweight or obesity or with existing metabolic abnormalities; however, these benefits may not translate to those with an already favorable cardiometabolic health status. Regardless of the potential misperceptions, the primary goal of utilizing the KD for endurance athletes is to gain a competitive advantage in competition, and combined with the assumption that their training status offers a degree of protection against adverse health outcomes, endurance athletes may be less concerned with the influence of the KD on their cardiometabolic health; particularly their blood lipid profiles.

In fact, unfavorable blood lipid alterations in response to a prolonged KD have been recently demonstrated in endurance athletes [9, 10]. This is particularly concerning for endurance athletes choosing to employ this dietary strategy, as they might be at increased risk of atherogenesis, large-artery wall stiffening, and myocardial fibrosis from excessive and long-term training [11, 12] before considering the compounding effects of injurious blood lipid profiles. As such, and given the ambiguous evidence in support of performance or metabolic benefits [2, 13], endurance athletes employing a KD may unnecessarily/unintentionally be putting themselves at a greater risk for cardiovascular dysfunction; potentially negating the preferable cardiometabolic health status they achieve through training.

Although previous studies have investigated the effect of a KD on the blood lipid profiles of endurance athletes, the available literature remains limited in several areas. Specifically, the majority of prior investigations have: (i) been conducted under highly controlled conditions which may not translate to practice; (ii) not compared the effects of a KD to the athlete’s habitual diet (HD), which tends to align with the high-fat Western diets practiced by their non-athlete counterparts [1, 2], or the more recommended HCD; (iii) not investigated postprandial lipid responses to a corresponding KD meal despite that endurance athletes are continually in an interprandial state to accommodate fueling and recovery needs; and (iv) not shown the speed in which blood lipid alterations are developed through short-term interventions. Thus, the purpose of this study was to examine the chronic and postprandial blood lipid alterations following a two-week *ad libitum* KD compared to an *ad libitum* HCD and HD in trained competitive cyclists and triathletes.

## Methods

This study is a secondary analysis originating, but separate, from a larger line of investigation [1, 2, 14]. The primary outcome variables in the present study are distinct, with only the control variables and participant characteristics being shared. As such, a detailed description of the current study’s methodological approaches have been published elsewhere [1, 2, 14], but are summarized hereafter for clarity; with any additional information specific to the methods of this analysis provided in further detail. The prospective registration of this study is located at ClinicalTrials.gov Identifier: NCT04097171. This study adhered to all CONSORT guidelines. The original study protocol for this clinical trial, including the CONSORT diagram demonstrating participant flow, can be found in Graybeal et al. [1].

### Participants

Participant characteristics are presented in Table 1. Six trained competitive [15] cyclists and triathletes (females: 4, males: 2; age: 37.2 ± 12.2 years) completed the study in its entirety. Additional inclusion criteria included cycling at least 100 km/wk over the last year and a V̇O_2_max ≥ 80th percentile for their age and sex (46.6 ± 6.7 mL/kg/min). A complete list of the exclusion criteria are presented elsewhere (1).

**Table 1:** Participant Characteristics

*N* = 6	
Age (y)	37.2 ± 12.2
Height (cm)	172.3 ± 10.0
Weight (kg)	68.5 ± 17.5
BMI (kg/m^2^)	22.7 ± 3.4
V̇O2max (ml/kg/min)	46.6 ± 6.7
RMR (kcals/d)	1617.3 ± 314.7

Data are presented as mean ± standard deviation

BMI: body mass index; V̇O2max: relative maximal oxygen consumption; RMR: resting metabolic rate

The study was approved by the Texas Christian University Institutional Review Board (IRB #192 − 14) and conducted in accordance with the Declaration of Helsinki. Written informed consent was obtained from all participants.

### Procedures

For this randomized crossover trial, participants completed a pre-screening visit to verify eligibility (including V̇O_2_max testing) and to complete anthropometric assessments and measurements of resting metabolic rate (RMR); all of which are described in detail elsewhere (1). Following the screening visit, eligible participants were tested during their HD and again following both a 14-d KD and HCD; with the KD and HCD being assigned in a random order by an investigator using block randomization. Because ketosis can be achieved in as few as 3–5 d during the KD [5, 6] and be washed out immediately after commencement of a HCD [16], a defined washout period was not employed. Thus, after completing the first randomly assigned 14-d diet (i.e., the KD or HCD) participants were immediately crossed over to the other diet. In addition, the effects of a HCD can be rapidly removed after initiation of a KD given that severe carbohydrate restriction is trademark to the KD. Furthermore, capillary and urinary β-hydroxybutyrate (βHB) were ≥ 0.5 mmol/L by the midway point of the KD for participants transitioning from the HCD to the KD and there were no effects of diet order on any primary or secondary variable across investigations in this line of research [1, 2, 14]; including the present study. Fasting and postprandial blood lipids were collected at each testing visit for all conditions including the HD. To measure postprandial blood lipid responses following each diet, participants consumed a test meal corresponding with each diet's macronutrient content; following the HD, participants consumed a test meal reflective of a traditional Western diet (16). Fasting measures were collected at baseline after a ≥ 12 h overnight fast from food, beverages other than water, supplements and medications, and a ≥ 24 h abstention from exercise; all of which were verified by an investigator prior to testing and conducted in the Exercise Physiology Laboratory at Texas Christian University. Postprandial blood lipid responses were measured at 30, 60, 120, and 180 min after ingesting each of the aforementioned test meals.

### Diet conditions and test meals

For the HD, participants were told to continue their normal dietary patterns *ad libitum*. Both the KD and HCD were followed *ad libitum*, and participants were instructed to consume 15% of their total daily energy from protein for each of these two diets. For the KD, participants were instructed to eat < 10% of their total daily energy from carbohydrates with the remainder of their total energy coming from dietary fat. For the HCD only, participants were required to consume ≥ 65% of their total daily energy from carbohydrates and < 20% from dietary fat.

Three-day dietary records (two weekdays and one-weekend day) were completed during the second week of each 14-d diet and were used to determine compliance to the KD and HCD. Diet records were also completed on three days in the week prior to testing for the HD. To ensure daily adherence to the KD and HCD, participants completed daily digital food logs and recorded their weight from a personal scale, both of which were sent to a registered dietitian (an investigator) for feedback. None of the participants exhibited weight fluctuations of ≥ ± 5%.

Adherence to the KD was also confirmed by both urinary and capillary βHB concentrations of ≥ 0.5 mmol/L; where daily adherence was verified by daily urinary ketone strips (1.82 ± 0.52 mmols/L) and general adherence was assessed by measuring capillary βHB at the midway (day 7) and endpoints (day 14) of the KD (0.99 ± 0.25 mmols/L). Participants were instructed to maintain their habitual physical activity and training habits for the length of the study which was evaluated via self-reported exercise logs and verified by an investigator [1]. In summary, energy intake and daily exercise characteristics (distance and session rating of perceived exertion) were not different across conditions [1]. While weight was significantly lower following the KD compared to the HD and HCD, energy intake was highest for the KD (non-significant) and weight fluctuations were < 5.0% across conditions. Although objective energy expenditure data was not collected to confirm the eucaloric design of the diet conditions, the estimated total daily energy needs (RMR multiplied by an activity factor of 1.6) across participants were 2,587.7 ± 503.5 kcals/d; markedly similar to the energy intake during both the KD (2,447.2 ± 195.8 kcals/d) and HCD (2,418.4 ± 266.3 kcals/d).

For the KD, carbohydrate intake (8.7 ± 2.9%) was significantly lower and fat intake (64.1 ± 5.39%) was significantly higher during the KD compared to the HCD (carbohydrate: 63.3 ± 8.8%; fat: 20.8 ± 7.6%) and the HD (carbohydrates: 45.8 ± 6.86%; fat: 38.2 ± 7.8%) and ketosis was verified throughout the KD using βHB concentrations (day 7 and day 14) and daily urinary ketones. For the HCD, carbohydrate intake was significantly higher and fat significantly lower when compared to the HD. Protein intake was significantly higher during the KD (26.0 ± 2.9%) compared to the HCD (14.4 ± 3.2%) and HD (16.5 ± 4.2%) with no differences in protein intake between the HCD and the HD. Employing the same repeated measures techniques described below, saturated fatty acid intake (as % of total energy intake) was significantly higher (*p* = 0.006) during the KD (23.8 ± 4.7%) compared to the HCD (5.5 ± 2.2%; *p* < 0.001) and HD (10.7 ± 2.8%; *p* < 0.001), and during the HD when compared to the HCD (*p* = 0.018).

The recipes used for each test meal are described in detail elsewhere [1]. In summary, the energy, protein (~ 15%), volume, and fiber content were the same across test meals with the only differences being the carbohydrate and fat content provided from each separate test meal. The energy content (kcals) of the test meal consisted of 60% of the participants RMR collected at screening. The carbohydrate and fat content of the KD and HCD test meals were in accordance with the aforementioned percentages of each diet, and the contents of the HD were in consort with a standard Western diet [17].

### Blood lipid biomarker analysis

Serum blood lipids including total cholesterol (TC), triglycerides (TRG), high-density lipoprotein cholesterol (HDL-C), non-HDL-C, and low-density lipoprotein cholesterol (LDL-C) were collected at baseline (fasting) after each diet (including the HD) and at 30, 60, 120, and 180 min postprandial following consumption of each diet’s corresponding test meal. Very low-density lipoprotein cholesterol (VLDL-C) was calculated as 20% of TRG. Other calculated blood lipid variables included %HDL-C (as a % of TC), TC:HDL, LDL:HDL, and TRG:HDL. Additionally, the blood lipid subfractions LDL-III (LDL_III_), LDL-IV (LDL_IV_), and HDL-IIb (HDL_IIb)_, and the blood lipid particle numbers for VLDL (VLDL_P_), LDL (LDL_P_), non-HDL (non-HDL_P_), HDL (HDL_P_), and remnant-like lipoprotein (RLP_P_) were measured at each timepoint. All blood lipids were analyzed by BioReference Laboratories Inc. (Elmwood Park, NJ).

### Statistical analysis

Fasting blood lipids following each diet condition were assessed using Friedman tests with Durbin-Conover post hoc analyses. For postprandial blood lipid responses following the consumption of each diet’s corresponding test meal, incremental area under the curve (iAUC) and peak postprandial responses (defined as the highest recorded value of each variable after baseline) were determined for each blood biomarker; each of which were assessed across condition using Friedman tests with Durbin-Conover post hoc analyses. Significance was determined at *p* < 0.050. The effect of diet condition order were assessed using Friedman tests and revealed no significant effect of diet condition order on any fasting blood lipid biomarker. Missing blood lipid data were missing completely at random and handled using linear regression. Statistical analyses were conducted using R version 4.1 [18] which included the PMCMR package [19].

## Results

### Chronic alterations in blood lipids

Fasting blood lipid measurements collected at the end of each diet condition are presented in Table 2. There were significant effects of diet condition on TC (*p* = 0.015), with post hoc tests revealing that TC was significantly higher following the KD compared to the HD (*p* < 0.001) and the HCD (*p* = 0.006). There were no significant differences in TC between the HD and HCD (*p* = 0.245), and no other significant effects of diet condition.

**Table 2:** Fasting blood lipids in response to an ad libitum two-week KD, HCD, and the participants’ HD

*N* = 6	HD	HCD	KD
	Mean (95%CI)	Mean (95%CI)	Mean (95%CI)
TC (mg/dL)	158.7 (128.4, 189.0)	163.5 (129.7, 197.3)	188.3^†#^ (151.0, 225.7)
TRG (mg/dL)	64.8 (29.1, 100.5)	77.4 (44.4, 110.4)	61.7 (44.7, 78.7)
HDL-C (mg/dL)	66.3 (52.9, 79.8)	63.9 (53.0, 74.8)	73.2 (56.9, 89.5)
HDL%	43.2 (31.7, 54.7)	41.0 (28.6, 53.4)	40.0 (28.8, 51.2)
HDL_P_ (nmol/L)	7426 (6305, 8547)	7695 (6770, 8620)	7522 (6423, 8621)
HDL_IIb_ (nmol/L)	2905 (2245, 3565)	2803 (2168, 3438)	2897 (2223, 3572)
LDL-C (mg/dL)	79.5 (52.3, 106.7)	84.0 (48.7, 119.3)	102.8 (61.6, 144.1)
LDL_P_ (nmol/L)	572.2 (400.7, 743.7)	560.5 (448.2, 672.9)	695.5 (472.6, 918.5)
LDL_III_ (nmol/L)	130.7 (67.6, 193.8)	176.7 (44.4, 309.0)	188.2 (70.9, 305.4)
LDL_IV_ (nmol/L)	134.0 (93.6, 174.4)	117.0 (81.2, 152.9)	136.7 (87.7, 185.6)
VLDL-C (mg/dL)	12.8 (5.6, 20.0)	15.6 (8.8, 22.3)	12.3 (8.8, 15.8)
VLDL_P_ (nmol/L)	10.7 (1.6, 19.7)	16.5 (8.2, 24.8)	10.6 (3.0, 18.1)
RLP (nmol/L)	59.3 (28.5, 90.2)	63.8 (35.9, 91.7)	74.7 (22.8, 126.6)
Non-HDL-C (mg/dL)	92.3 (59.0, 125.7)	99.6 (57.8, 141.3)	115.2 (71.1, 159.2)
Non-HDL_P_ (nmol/L)	582.0 (405.2, 758.8)	577.0 (462.8, 691.2)	705.0 (474.3, 935.7)
LDL:HDL	1.28 (0.55, 2.01)	1.43 (0.47, 2.38)	1.56 (0.48, 2.63)
TC:HDL	2.50 (1.59, 3.41)	2.69 (1.55, 3.83)	2.73 (1.60, 3.87)
TRG:HDL	1.10 (0.11, 2.07)	1.32 (0.46, 2.19)	0.90 (0.43, 1.37)

HD: habitual diet; HCD: high-carbohydrate diet; KD: ketogenic diet; HDL: high density lipoprotein cholesterol; HDL_P_: high density lipoprotein particle number; LDL: low-density lipoprotein cholesterol; LDL_P_: low-density lipoprotein cholesterol particle number; RLP_P_: remnant-like lipoprotein particle number; TRG: triglycerides; VLDL: very low-density lipoprotein cholesterol; VLDL_P_: very low-density lipoprotein cholesterol particle number

# significantly different from the habitual diet at *p* < 0.050

† significantly different from the high-carbohydrate diet at *p* < 0.050

### Postprandial blood lipid responses

Postprandial iAUC for each blood lipid measurement following the ingestion of each test meal are shown in Table 3. There were significant effects of test meal on TRG_iAUC_, TRG:HDL_iAUC_, and VLDL_iAUC_ (all *p* = 0.009) with post hoc tests revealing that TRG_iAUC_, TRG:HDL_iAUC_, and VLDL_iAUC_ were all significantly higher following consumption of the KD test meal compared to both the HD (all *p* < 0.001) and the HCD test meals (all *p* = 0.001). Significant effects of test meal were also observed for LDL_iAUC_ (*p* = 0.011) and LDL:HDL_iAUC_ (*p* = 0.006), each of which was significantly lower following the KD compared to both the HD and HCD test meals (all *p* < 0.001).

**Table 3:** Total and peak postprandial blood lipid responses to a KD test meal, a HCD test meal, and a test meal reflecting the participants’ HD

	*N* = 6	HD	HCD	KD
		Mean (95%CI)	Mean (95%CI)	Mean (95%CI)
iAUC	TC (mg/dL)	3.5 (-26.6, 33.6)	11.0 (-20.5, 42.5)	6.7 (-40.0, 53.3)
	TRG (mg/dL)	102.8 (27.7, 178.0)	120.4 (74.7, 166.2)	286.2 (175.6, 396.7)^†#^
	HDL-C (mg/dL)	-5.8 (-14.1, 2.5)	1.1 (-13.6, 15.7)	-7.50 (-19.7, 4.7)
	HDL-C% (%Δ)	-3.8 (-7.7, 0.0)	-2.6 (-6.7, 1.4)	-5.00 (-11.7, 1.7)
	HDL_P_ (nmol/L)	2429 (-2171, 7029)	3557 (271, 6843)	966 (-2854, 4786)
	HDL_IIb_ (nmol/L)	-81 (-2455, 2294)	346(-1233, 1925)	822 (-887, 2530)
	LDL-C (mg/dL)	-12.0 (-28.9, 4.9)	-14.0 (-34.7, 6.7)	-43.2 (-65.1, -21.3)^†#^
	LDL_P_ (nmol/L)	344.2 (-320.4, 1008.8)	232.3 (-449.3, 913.9)	370.2 (-224.4, 964.8)
	LDL_III_ (nmol/L)	165.2 (-187.9, 518.2)	-30.9 (-276.8, 214.9)	132.7 (-66.9, 332.2)
	LDL_IV_ (nmol/L)	-8.0 (-174.7, 158.7)	50.0 (-59.2, 159.2)	102.7 (-78.2, 283.5)
	VLDL-C (mg/dL)	21.3 (6.2, 36.5)	23.9 (14.9, 32.9)	57.3 (35.1, 79.6)^†#^
	VLDL_P_ (nmol/L)	25.1 (-19.6, 69.8)	11.1 (-20.3, 42.4)	33.3 (-0.6, 67.2)
	RLP (nmol/L)	46.8 (-46.5, 140.2)	76.9 (-29.6, 183.5)	39.7 (-30.2, 109.5)
	Non-HDL-C (mg/dL)	9.3 (-13.4, 32.1)	10.0 (-8.1, 28.0)	14.2 (-22.1, 50.5)
	Non-HDL_P_ (nmol/L)	303.5 (-265.5, 872.6)	242.7 (-464.3, 949.6)	340.8 (-283.9, 965.4)
	LDL:HDL	-0.12 (-0.28, 0.05)	-0.23 (-0.38, -0.09)	-0.52 (-0.75, -0.28)^†#^
	TC:HDL	0.30 (-0.10, 0.70)	0.19 (-0.12, 0.50)	0.38 (-0.01, 0.78)
	TRG:HDL	1.90 (0.18, 3.62)	2.0 (0.41, 3.69)	4.35 (2.54, 6.16)^†#^
Peak	TC (mg/dL)	163.2 (128.6, 197.8)	171.8 (137.2, 206.5)	195.3 (155.3, 235.4)^†#^
	TRG (mg/dL)	113.7 (30.8, 196.6)	133.2 (78.0, 188.3)	191.0 (123.9, 258.1)
	HDL-C (mg/dL)	66.50 (53.4, 79.6)	66.83 (55.2, 78.5)	75.33 (58.5, 92.2)^†#^
	HDL-C% (%Δ)	43.00 (31.0, 55.0)	41.50 (29.0, 54.0)	40.50 (29.5, 51.5)
	HDL_P_ (nmol/L)	8687 (7579, 9795)	9126 (8641, 9610)	8587 (8296, 8878)
	HDL_IIb_ (nmol/L)	3244 (2787, 3701)	3198 (2606, 3789)	3408 (2964, 3851)
	LDL-C (mg/dL)	80.8 (49.9, 111.7)	83.8 (50.6, 117.1)	94.2 (54.7, 133.7)
	LDL_P_ (nmol/L)	904.5 (614.8, 1194.2)^†^	708.8 (473.1, 944.6)	937.4 (617.1, 1257.6)^†^
	LDL_III_ (nmol/L)	229.7 (95.6, 363.8)^†^	199.7 (67.8, 331.6)	283.4 (121.2, 445.5)^†^
	LDL_IV_ (nmol/L)	161.7 (143.7, 179.6)	146.8 (127.0, 166.6)	177.7 (136.2, 219.2)
	VLDL-C (mg/dL)	22.8 (6.3, 39.4)	26.7 (15.4, 37.9)	38.2 (24.6, 51.7)
	VLDL_P_ (nmol/L)	30.7 (-11.8, 73.1)	22.9 (10.4, 35.4)	31.8 (5.5, 58.1)
	RLP (nmol/L)	93.7 (35.8, 151.5)	96.3 (51.9, 140.7)	112.0 (33.7, 190.3)
	Non-HDL-C (mg/dL)	97.7 (59.9, 135.4)	106.2 (62.5, 149.9)	124.3 (80.4, 168.3)
	Non-HDL_P_ (nmol/L)	868.0 (656.7, 1079.4)	729.7 (481.8, 977.5)	900.5 (655.1, 1145.9)
	LDL:HDL	1.32 (0.55, 2.08)	1.41 (0.47, 2.34)	1.53 (0.50, 2.56)
	TC:HDL	2.65 (1.62, 3.68)	2.85 (1.56, 4.15)	2.95 (1.72, 4.18)
	TRG:HDL	2.02 (-0.11, 4.16)	2.39 (0.59, 4.19)	2.86 (1.65, 4.07)

iAUC: incremental area under the curve; HD: habitual diet; HCD: high-carbohydrate diet; KD: ketogenic diet; HDL-C: high density lipoprotein cholesterol; HDL_P_: high density lipoprotein particle number; LDL-C: low-density lipoprotein cholesterol; LDL_P_: low-density lipoprotein cholesterol particle number; RLP_P_: remnant-like lipoprotein particle number; TRG: triglycerides; VLDL-C: very low-density lipoprotein cholesterol; VLDL_P_: very low-density lipoprotein cholesterol particle number

# significantly different from the habitual diet at *p* < 0.050

† significantly different from the high-carbohydrate diet at *p* < 0.050

Peak postprandial concentrations of each blood lipid marker after baseline are presented in Table 3. TC_peak_ (*p* = 0.030) and HDL_peak_ (*p* = 0.042) were significantly different by test meal, where TC_peak_ and HDL_peak_ were significantly higher following the KD test meal compared to both the HD (TC_peak_: *p* = 0.004; HDL_peak_: *p* = 0.023) and HCD (TC_peak_: *p* = 0.044; HDL_peak_: *p* = 0.012) test meals. LDL_P−peak_ (*p* = 0.011) and LDL_III−peak_ (*p* = 0.015) were significantly different by test meal, with post hoc tests revealing that LDL_P−peak_ and LDL_III−peak_ were significantly lower following the HCD test meal compared to the HD (LDL_P−peak_: *p* < 0.001; LDL_III−peak_: *p* = 0.006) and KD (both *p* < 0.001) test meals.

## Discussion

This study sought to determine the chronic and postprandial blood lipid alterations in a group of trained competitive cyclists and triathletes following a two-week *ad libitum* KD when compared to a HCD and HD. Although several studies have examined the changes in blood lipid profiles following a KD in various groups of trained athletes [9, 10, 20–25], fewer have evaluated the KD *ad libitum* [10, 20–22, 24], and the majority have compared the KD to a HCD [9, 10, 20–24], or a HD [21, 25], but not both. As such, our approach allowed us to evaluate diet-induced changes in blood lipids (i) under more practical circumstances—demonstrating the responses more likely to be observed outside of a laboratory environment; and (ii) when compared to the HCD recommended by most professional organizations [26] and the athletes’ typical dietary patterns —generally more reflective of a high-fat Western diet [2]. To our knowledge, our study may be the first to examine the postprandial blood lipid responses to a KD test meal during a KD in endurance athletes, as most studies have evaluated blood lipid shifts in response to exercise [22, 24, 25], despite that athletes are in the interprandial state more often than training [2]. Overall, the primary finding of our study was that a two-week KD resulted in higher TC compared to a HCD and the athlete’s HD. While we observed lower postprandial LDL-C and LDL:HDL, postprandial TRG, TRG:HDL, and VLDL-C were higher following the KD test meal compared to the other diet conditions.

In accordance with our findings, prior studies have reported chronic hypercholesterolemia following a KD in trained athletes [9, 10, 22, 23, 25], with some (similar to our study) observing increases in TC after 14-d [23], highlighting the rapid nature of these alterations following this extreme dietary shift. Under normal circumstances, aerobic exercise at higher training intensities alters blood lipids by directly increasing HDL-C and decreasing LDL-C [27]. However, our finding of higher TC following the KD was a product of simultaneous increases (albeit non-significant) in HDL-C and LDL-C as previously reported [9, 23, 25]. This is noteworthy considering that higher HDL-C concentrations would typically stimulate LDL-C removal with additional reductions in LDL-C expected to occur from the training practices of our sample. However, not only did we observe significantly higher TC following the KD without differences in HDL-C or LDL-C between diet conditions, we did not observe lower TRG which has been frequently reported for KD [20, 23, 28]. Our previous work in this line of investigation has shown that fat oxidation is similar between a KD and HD; where athlete’s may demonstrate a degree of “fat adaptation” (or tolerance) as a result of their higher-fat HD. Yet, TC was significantly higher following KD in our study despite continual exposure to the high-fat intakes of their HD patterns. We suggest several potential explanations below.

Our findings conflict with the findings of other studies that report improvements in cardiometabolic profiles following a KD when compared to an HCD. This is likely because the KD is predominately used as a weight-loss intervention for individuals with existing cardiometabolic abnormalities [29, 30], with few studies examining the KD under eucaloric conditions. As such, our eucaloric design, evident by weight maintenance and negligible differences between estimated total daily energy needs and verified energy intake, may explain the differences between our findings and others, given that the greatest changes in TC for both trained and untrained individuals are observed when energy balance during a KD is maintained [31]. Moreover, and given that studies showing improvements in blood lipids following a KD are often conducted at lower absolute energy intakes, the higher maintenance energy needs of endurance athletes are worth noting, as the energy from fat necessary to facilitate weight maintenance during intensive training periods are expected to be considerably higher than the fat intakes of hypocaloric diets. Interestingly, the athletes in our study did experience significantly greater weight loss during KD, and we were able to observe higher TC during the KD despite the well-reported effects of weight loss on circulating blood lipids [32]. However, this could be considered speculative, as weight loss was modest and likely a product of losses in glycogen and total body water [16]. While we did monitor physical activity and training throughout the intervention, it is common for athletes following the KD to experience fatigue and increased perception of effort during training [13, 33]. As such, athletes in our study may have felt they were giving greater training efforts during the KD (i.e., increased perceptual effort), but were undergoing passive declines in objective training intensity (i.e., lower power output). This may have blunted any exercised-induced increases in HDL-C or decreases in LDL-C that they may have experienced under normal circumstances; although it could also be that the two-week intervention was too short in duration to observe these changes.

Although studies have consistently shown increases in HDL-C following a KD, these increases are relatively modest; often showing an inability to overcome the simultaneous increases in LDL-C that occur when large quantities of dietary fat are rapidly introduced. This may be particularly true for endurance athletes who typically demonstrate higher HDL-C from favorable lifestyle behaviors and consistent aerobic training. It is possible that differences in fatty acid composition explains our findings of higher TC, as the KD is inherently higher in saturated fatty acids (SFA) which largely contributes to increases in LDL-C and TC. In fact, Burén and colleagues [34] showed that in a sample of normal-weight young healthy females, a eucaloric KD with ~ 33% energy from SFA resulted in significantly higher TC, LDL-C, and small dense LDL subfractions, and lower LDL particle size; similar to our postprandial results showing significantly higher LDL_Ppeak_ and LDL_IIIpeak_ following the KD. Additionally, Creighton et al. [9] also found higher TC, LDL-C, and LDL particle number in a group of keto-adapted endurance athletes consuming ~ 27% of their total energy from SFA when compared to athletes participating in a HCD. Although unsurprising, the findings from the aforementioned studies were observed despite increases in HDL-C; further supporting the inability of HDL-C to facilitate the removal of LDL-C during a KD. While a low-SFA KD could be proposed, this is typically less feasible in practice, as athletes may have difficulty reaching the proposed fat intakes of a KD. Thus, athlete’s may be more likely to resort to more convenient fat sources (which are often higher in both total fat and SFA) to meet the dietary fat demands of a KD.

Coupled with elevated fat intake, the KD is fundamentally lower in fiber. Bile acids, which are important lipid emulsifiers synthesized from cholesterol, undergo elevated synthesis and release in response to higher fat intakes [35]. After facilitating lipid absorption in the small intestine, bile acids are reabsorbed and transported back into hepatic tissue. Fiber, however, prevents bile acid reabsorption, stimulating the removal of cholesterol from the plasma (i.e., lowering cholesterol) to resynthesize bile acids in the liver [36]. Nevertheless, without sufficient fiber intake from severe carbohydrate restriction, bile acid reabsorption suppresses the transportation of cholesterol to liver, particularly LDL-C, potentially contributing to the higher TC observed during a KD.

Although the majority of available literature regarding the relationship between KDs and blood lipids are based on findings in the fasted state [37], humans spend the majority of their waking hours in the interprandial state, where additional food and beverage are consumed before circulating lipids return to pre-prandial levels [38]. Because the postprandial state is more translatable to practice and demonstrates more dynamic shifts in lipid metabolism relative to the fasted state [38], examining postprandial lipid alterations after the consumption of a KD-type meal, whilst in a ketogenic state, may more effectively highlight the changes in circulating lipids expected to occur in practice. To that end, we observed significantly higher postprandial iAUC responses for TRG, TRG:HDL, VLDL-C, in addition to significantly lower postprandial iAUC responses for LDL-C and LDL:HDL following the consumption of a KD meal compared to the other conditions. Is has been postulated that endurance athletes demonstrate greater metabolic flexibility relative to their untrained counterparts, which refers to an individual’s ability to match fat oxidization to fat availability [2, 39]. The increased metabolic flexibility of endurance athletes is supported by studies showing lower fasting TRG during eucaloric KDs. However, our findings are unique, showing that TRGs remain elevated following the consumption of a KD meal under ketogenic conditions compared to the other postprandial conditions. Given our collective postprandial findings, the metabolic flexibility of endurance athletes may be subjected to a potential ceiling effect; where fat oxidation eventually plateaus despite increasing fat availability. It is important to note, however, that LDL-C responses were lower following the consumption of a KD meal. It could be that the higher fat content within the KD meal stimulated bile acid synthesis, requiring LDL-C extraction from the plasma to the liver (leading to lower postprandial plasma concentrations), or that the higher peak HDL-C facilitated LDL-C removal. However, it remains unknown if these dynamic changes manifest in chronic lipid alterations and thus, further research is necessary.

A complete list of the limitations within this line of investigation have been described in detail elsewhere [1, 2, 14] but are briefly described. The primary limitation was that the COVID-19 pandemic resulted in the premature stoppage of our study, leading to a smaller sample size than originally anticipated. However, using the achieved effect size (f = 1.47) for our primary variables of interest (TC) and with a conservative correlation of *r* = 0.30, our achieved power analysis indicated that six participants would produce 98.6% power at an α = 0.05, and we were able to observe several other significant findings. Nevertheless, large effect sizes produced from a small sample should be interpreted with caution. The length of each intervention may also be considered as a limitation, but several studies have reported that ketosis can be achieved within 5-d of a KD [6] and others have reported blood lipid changes within two-weeks of a KD [23]. The *ad libitum* nature of the KD in our study also led to higher protein intake than expected. However, given the difficulty in consuming the fat intakes required for the KD, it is likely that this better represents the composition of the KD in practice.

In conclusion, trained competitive cyclists and triathletes may demonstrate increased TC in response to a 14-d KD compared to a HCD or the athletes’ HD. Further, the consumption of a KD test meal during a KD may result in higher postprandial responses for TRG, TRG:HDL, and VLDL-C, and higher postprandial peaks for TC and HDL-C; but may also result in lower postprandial responses for LDL-C and LDL:HDL. Given the ambiguous findings for endurance performance, trained competitive cyclists and triathletes contemplating a KD strategy should consider the potential for these blood lipid alterations. Future research should consider a focus on postprandial blood lipid alterations to determine if this more dynamic measurement manifests in chronic blood lipid changes in this group.

## Data Availability

Data is available upon reasonable request. Please contact Austin J. Graybeal, PhD, CSCS, Assistant Professor, School of Kinesiology & Nutrition, College of Education and Human Sciences, University of Southern Mississippi, Hattiesburg, MS 39406, USA. Phone: 601-266-5996. Email address: austin.graybeal@usm.edu.

## Abbreviations

βHB: Beta-hydroxybutyrate
HCD: High-carbohydrate diet
HD: Habitual diet
HDL-C: High density lipoprotein cholesterol
HDL_P_: High density lipoprotein particle number
iAUC: Incremental area under the curve
KD: Ketogenic diet
LDL-C: Low-density lipoprotein cholesterol
LDL_P_: Low-density lipoprotein cholesterol particle number
PMCMR: Pairwise multiple comparisons of mean rank sums
RLP_P_: Remnant-like lipoprotein particle number
RMR: Resting metabolic rate
SFA: Saturated fatty acids
TC: Total cholesterol
TRG: Triglycerides
VLDL-C: Very low-density lipoprotein cholesterol
VLDL_P_: Very low-density lipoprotein cholesterol particle number
V̇O2max: Maximal relative oxygen consumption
